# A facile strategy for rapid preparation of graphene spongy balls

**DOI:** 10.1038/srep32746

**Published:** 2016-09-02

**Authors:** Shu Wan, Hengchang Bi, Xiao Xie, Shi Su, Kai Du, Haiyang Jia, Tao Xu, Longbing He, Kuibo Yin, Litao Sun

**Affiliations:** 1 SEU-FEI Nano-Pico Center, Key Laboratory of MEMS of Ministry of Education, Collaborative Innovation Center for Micro/Nano Fabrication, Device and System, Southeast University, Nanjing 210096, P. R. China; 2 Center for Advanced Carbon Materials, Southeast University and Jiangnan Graphene Research Institute, Changzhou 213100, P. R. China; 3 Southeast University-Monash University Joint Research Institute, Suzhou 215123, P. R. China

## Abstract

Porous three dimensional (3D) graphene macrostructures have demonstrated the potential in versatile applications in recent years, including energy storage, sensors, and environment protection, etc. However, great research attention has been focused on the optimization of the structure and properties of graphene-based materials. Comparatively, there are less reports on how to shape 3D graphene macrostructures rapidly and effortlessly, which is critical for mass production in industry. Here, we introduce a facile and efficient method, low temperature frying to form graphene-based spongy balls in liquid nitrogen with a yield of ~400 balls min^−1^. Moreover, the fabrication process can be easily accelerated by using multi pipettes working at the same time. The graphene spongy balls show energy storage with a specific capacitance of 124 F g^−1^ and oil adsorbing with a capacity of 105.4 times its own weight. This strategy can be a feasible approach to overcome the low efficiency in production and speed up the development of porous 3D graphene-based macrostructures in industrial applications.

Since great progress of graphene synthesis techniques has been made in recent years^1^,^2^,^3^, massive production of graphene nanosheets with desirable properties have been emerged. The graphene nanosheets have shown potential in various kinds of applications, such as supercapacitors^4^, electrodes in lithium battery^5^, adsorbents^6^,^7^, separative membranes^8^ and sensors^9^. However, it is essential to assemble them into three dimensional (3D) macrostructures to meet the requirements in real-life applications. The applications of graphene supercapacitor electrodes^10^,^11^,^12^ and scaffold for photocatalystic activities^13^ have proven that 3D porous graphene macrostructures via assembly of nanosized graphene sheets are promising for industry. The success could be attributed to the good conductivity and high surface area of graphene material. Therefore, to develop efficient production strategies becomes a critical challenge in order to construct 3D monoliths facilely, massively and controllably^14^.

Unlike other well-established methods^15^,^16^,^17^, assembling graphene nanosheets into membranes, creating blocks and spheres like thicker 3D structures seems more difficult. The major obstacle is the easy aggregation of graphene nanosheets during the assembling process, resulting in a significant decrease of surface-to-volume ratio and then deteriorating the properties of graphene. Therefore, how to overcome the aggregation problem during the formation of 3D graphene macrostructures draws the attention from researchers. Owing to the good dispersability in water, graphene oxide (GO), the most important derivative of graphene, becomes one of the building blocks to realize the construction of graphene 3D frameworks. Previous reports have proven that hydrothermal treatment for GO dispersion can be an alternative to construct various kinds of porous graphene 3D macrostructures^18^,^19^,^20^. Niu *et al.*^21^ reported a leavening process, demonstrating that a foam-like 3D framework could be inflated from GO films with a tightly packed structure through gas releasing during GO reduction. In addition, some researchers have investigated the inclusion of spacers to enlarge GO layer spacing by introducing polymer beads or nanoparticles as nanofillers^22^,^23^. Those developments have explored new directions to achieve various kinds of graphene-based macrostructures with expected properties for a variety of applications such as biochemical sensors, energy storage and environment protection. However, despite the advantages of previous methods, they leave the common challenge to massive, continuous synthesis of 3D graphene frameworks, due to their complicated and manpower-consuming fabrication process, which will strongly hinder the development in real life applications.

Here we report a novel assembling strategy aiming to realize rapid, continuous production of 3D graphene macrostructures through a liquid nitrogen-aided frying strategy. By dripping the GO dispersion drops directly into liquid nitrogen through a pipette, frozen GO balls of several millimeters in diameter can be continuously synthesized. In a typical experiment, the dripping speed is 0.5 mL s^−1^ per pipette, which can be further accelerated easily by multi-pipettes. Followed by freezing desiccation and chemical reduction, the graphene spongy balls (GSB) can be obtained. Due to the electrically conductive and the porous nature of GSB, they have been applied as electrodes for supercapacitor as well as efficient adsorbents for oils and organics. The GSB-based supercapacitor has shown a high specific capacitance of 124 F g^−1^. On the other hand, GSB showed an oil-adsorbing capacity as high as 105.4 times its own weight. Based on these promising properties, the present production strategy can be developed to an efficient technique to meet the criteria of labor-saving, massive and low-cost production in industrial practice.

## Results

### Massive production of GSB

**Figure 1:**
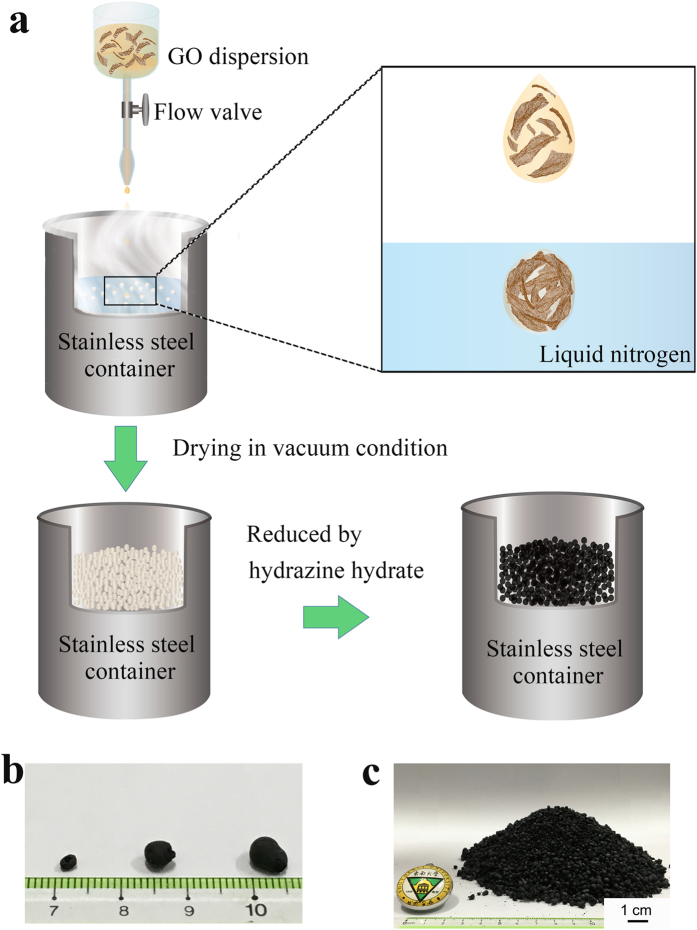
The production procedure of GSB. (**a**) The schematic illustration for the production of GSB. The frying process was shown in the zooming in view. A GO drop was frozen by liquid nitrogen. (**b**) The image of GSB with different sizes. (**c**) The image of a pile of GSB (~500 cm^3^) along with a badge of Southeast University (diameter: 3 cm).

The production procedure of GSB was schematically demonstrated in Fig. 1a. First, a drip pipette with tunable volume was adopted to pump the as-prepared graphene oxide (GO) dispersion (10 mg ml^−1^). The diameter of GSB could be controlled simply by varying the volume of each injection (Fig. 1b). Typically, the size of GSB could be modulated from hundreds of micrometers to millimeters by using this method. Then the GO drops were dripped into liquid nitrogen (−196 °C), which was stored in a thermal insulation container. Once GO drops (room temperature, 20 °C) contacted with liquid nitrogen, a rapid shaping of GO sheets happened due to the violent heat exchange (video 1 in Supporting Information). Either the apparent visual effect or the nature strong heat exchange, this process is similar to frying in the cooking technique. Therefore, graphene oxide frozen balls (GOFB) were successfully obtained. The flow velocity was 0.5 ml s^−1^ (~400 balls min^−1^), which means that 1 L GO dispersion can be fried by the liquid nitrogen within 30 min. It is noteworthy that the preparation time can be further reduced, thus the yield can be easily multiplied by applying multi-pipettes working at the same time. After the frying process, GOFB were freeze-dried along with the container for 12 hours. The resulting light brown, GO spongy balls (GOSB) were synthesized after the sublimation of both ice and liquid nitrogen. Finally, by using hydrazine hydrate as reduction agent, GOSB were chemically reduced into GSB, with their color turning to black. Besides the apparent change of the color, the sharp decrease of oxygen atomic concentration (from 29.33% to 9.64%, shown in Supporting Information Figs S1 and S2, measured by X-ray photoelectron spectroscopy) also indicated the elimination of oxygen-containing functional groups, such as hydroxyl- and carboxyl- groups. Moreover, the density of GSB was ranged from 1.8 mg cm^−3^ to 11 mg cm^−3^, by varying the concentration of GO dispersion. The results of GSB with different density were characterized by scanning electron microscopy (SEM), as shown in Supporting Information Fig. S3. Typically, particles with a denser structure exhibited better mechanical performance (Supporting Information Fig. S4). Figure 1c was a pile of GSB (~500 cm^3^) and showed the great potential for industrial production. Therefore, our strategy may serve as a promising candidate to fabricate rGO-based, 3D macrostructures with both dimension and density under precise control.

**Figure 2:**
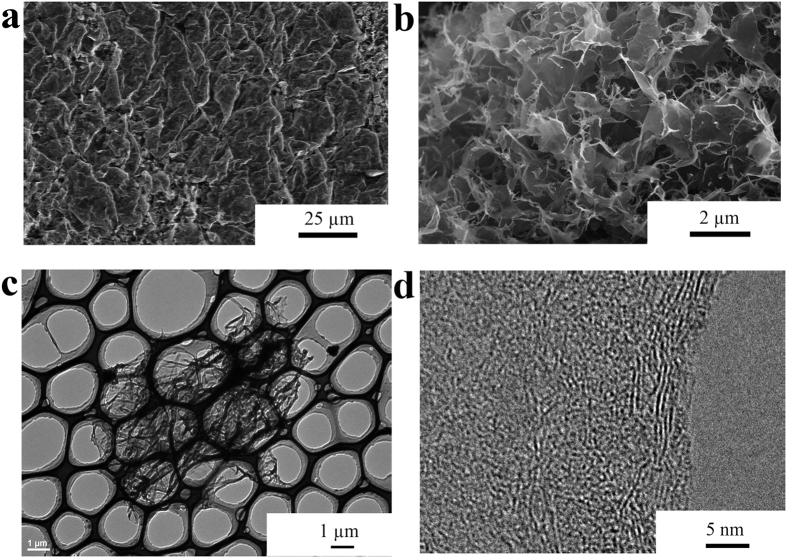
The microstructure of GSB. (**a**) The low magnification SEM image of GSB. (**b**) The high magnification SEM image of GSB. (**c**) The low magnification TEM image of GSB. (**d**) The high magnification TEM image of GSB.

SEM and transmission electron microscopy (TEM) were performed in order to depict the microstructure of GSB. The low-magnification structure was shown in Fig. 2a. GSB were constructed by fish-scale-like platelets with dimension from several to tens of micrometers. Detailed information could be found in higher magnifications (Fig. 2b). The porous structure of GSB was clearly observed and the average pore size was 1 ~ 2 μm. The graphene sheets, forming the wall of the pores, were quite thin (less than 10 layers) in TEM images (Fig. 2c,d). According to our previous report^24^, the pore size exhibited a strong dependence on the freezing temperature. Briefly, a lower freezing temperature would result in smaller pore size. The reason could be the ice tended to form smaller crystalline nuclei in low temperature. Therefore, varying the freezing temperature is an effective means to tailor the microstructure of GSB.

### Electrochemical performance of GSB

**Figure 3:**
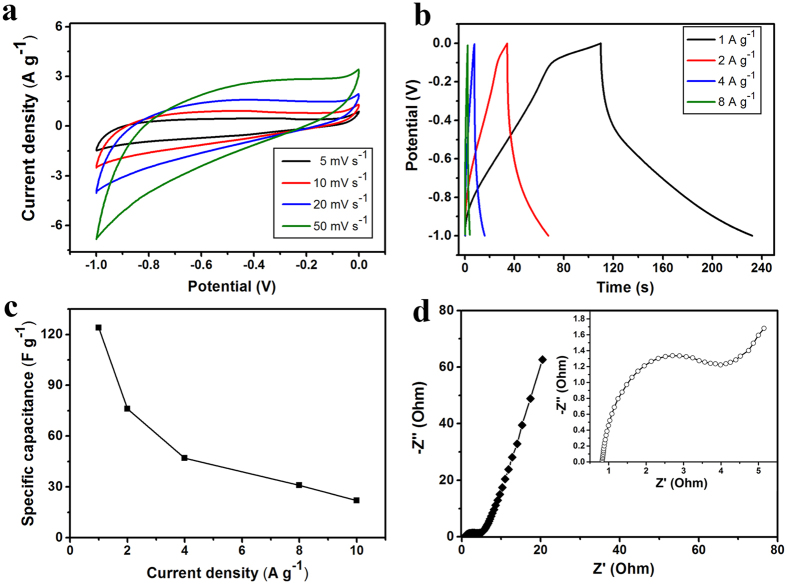
Performance of GSB-based supercapacitors. (**a**) The CV curves of GSB at different scan rates from 5 to 50 mV s^−1^. (**b**) Galvanostatic charge/discharge measurements of GSB at different current densities. (**c**) Specific capacitances of GSB at different current densities. (**d**) Electrochemical impedance spectroscopy of GSB at different frequencies, ranging from 0.01 Hz to 10 kHz.

The electrochemical performance of GSB (8 mg cm^−3^) electrodes was analyzed by cyclic voltammetry (CV) and galvanostatic charge/discharge measurements in 6 M KOH solution. In Fig. 3a, the potential window was set from −1.0 to 0 V with a scan rate from 5 to 50 mV s^−1^. The CV curves of GSB electrode showed roughly a rectangular shape without significant redox peaks, indicating a typical electrochemical double-layer capacitor (EDLC) behavior. Galvanostatic charge/discharge test was performed at different current densities from 1 to 8 A g^−1^ (Fig. 3b). As shown in Fig. 3c, the highest specific capacitance for GSB based on the charge/discharge curve is calculated as 124 F g^−1^ at a current density of 1 A g^−1^, which is comparable of previously reported rGO-based supercapacitors^25^,^26^. A detailed comparison of 3D state-of-the-art graphene based supercapacitor in recent years has been summarized in Table S1. The Nyquist plot of the electrodes was shown in Fig. 3d. Originating from the electrochemical impedance spectroscopy (EIS) in a frequency range from 0.01 Hz to 10 kHz, the plot demonstrated typical properties of an EDLC. The distinct semi-circle segment exhibited the characteristics of the bulk electrolyte resistance and charge transfer resistance. The short, 45° slope of Warburg segment at medium frequencies indicated the diffusion/transport of ions on the electrode surface or within the bulk of carbon electrode walls in the electrolyte. The power densities and energy densities of GSB-based symmetric supercapacitor were calculated and shown in Ragone plot (Fig. S5). The GSB symmetric supercapacitor delivered a power density of 5.46 kW kg^−1^ at an energy density of 2.4 Wh kg^−1^. Moreover, in order to evaluate the recycle ability of GSB supercapacitor, charging-discharging cycles were performed at 4 A g^−1^ (Fig. S6). ~93% of the initial capacitance was remained after 5000 cycles, demonstrating a long lifetime of the GSB supercapacitor. In the same current density, the coulombic efficiency was also measured and the value was around 90% (Fig. S6).

### Adsorption behaviors of GSB

**Figure 4:**
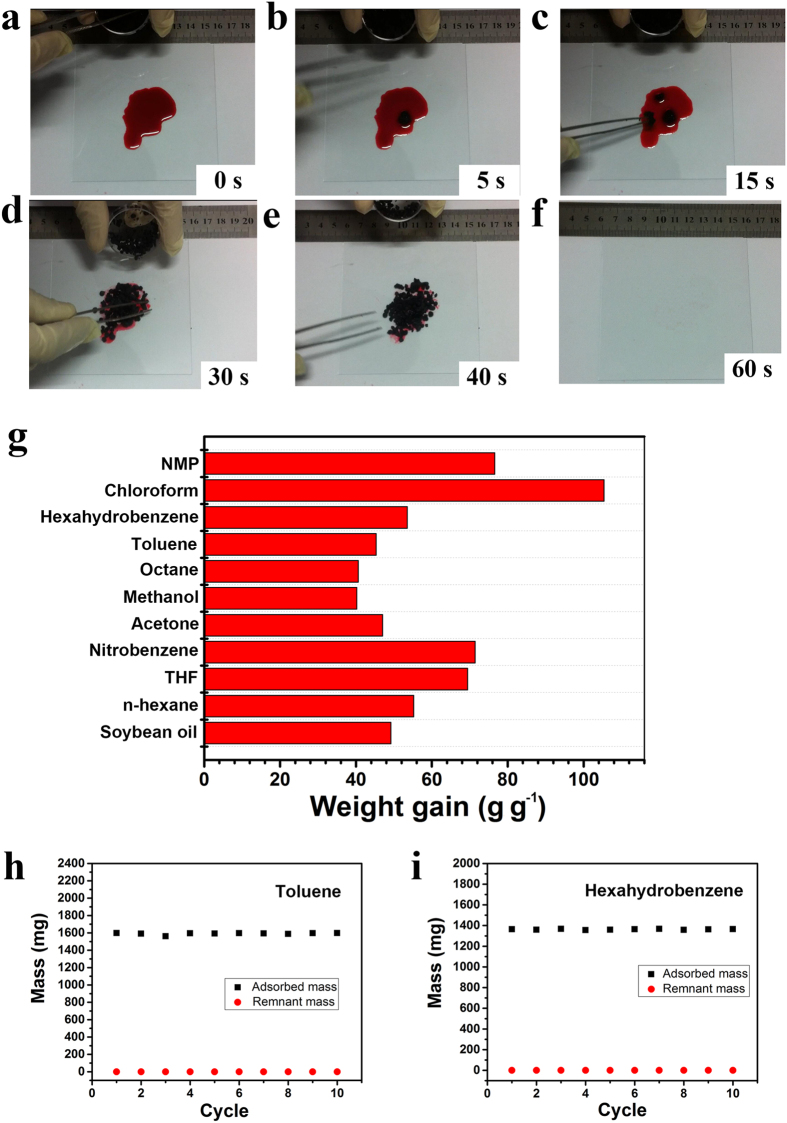
Adsorption capability and recyclability of GSB. (**a**–**f**) The adsorption process of hexahydrobenzene, stained by Sudan red 5B. The weights for hexahydrobenzene and GSB were ~1.5 g and 27 mg, respectively. (**g**) Adsorption capacity for various oils and organic solvents. (**h**,**i**) recyclability for toluene and hexahydrobenzene. (**h**): toluene. (**i**): hexahydrobenzene.

A series of adsorption experiments were then carried out to test the capability and recyclability of GSB (8 mg cm^−3^) for the adsorption of oils and organic solvents. The process of adsorption was shown in Fig. 4a–f. Once being placed on the top of a pool of hexahydrobenzene (~1.5 g, stained with Sudan red 5B), the GSB (~27 mg) rapidly adsorbed the hexahydrobenzene within 1 minute. Adsorption capacity of GSB was determined by their weight gain, which is defined as the weight of the adsorbed oil or solvent per unit weight of dried GSB. Here, GSB showed a high adsorption capacity towards various solvents, which was plotted in Fig. 4g. Among these solvents, the adsorption capacity for chloroform was the highest (105.4 times its own weight) and the overall adsorption capacities of GSB are higher than those of adsorbents prepared previously, e.g., by the leavening process^21^ and inorganic nanowire membranes^27^. Owing to the highly porous structure, GSB have strong affinity towards organic solvents and oils. This property, together with the simplicity of mass production endows GSB a suitable material to eliminate the crude oil spill, petroleum wastes, and toxic organic solvents^6^,^7^ in the practice of environmental protection.

In addition, as the pollutants are either precious raw materials or toxic solvents, it is also expected to recycle the adsorbed liquids and reuse the adsorbent in a proper means. Distillation is a straightforward method and was adopted to recycle the adsorbed oils and organics. Compared with other methods, such as solvent extraction or combustion, distillation is simple, highly efficient, labor-saving and inexpensive. The recyclability of GSB was tested by using toluene (b. p. 110.6 °C) and hexahydrobenzene (b. p. 80.7 °C). In a typical adsorption-desorption process, the heating temperature for vaporization was close to the boiling point of adsorbates. As an example, the GSB after adsorbing liquids were heated to 105 °C to release toluene vapor, or 75 °C to release hexahydrobenzene vapor. Then the heated GSB were weighed and compared with its original mass to determine the weight of ‘dead’ liquid resided in the adsorbent. The residual weights of both toluene and hexahydrobenzene in GSB were less than 3% for each adsorption-desorption cycle (Fig. 4h,i). As shown in Fig. 4h,i, little deterioration in adsorption capacity could be observed after 10 cycles, indicating an excellent recycling performance of GSB. The adsorbed organics were released by heating the sample followed by collecting the condensate by using a typical distillation experiment setup, which was schematically shown in Supporting Information Fig. S7. No physical damage to the GSB microstructure and nanostructure was observed according to SEM image, demonstrated in Supporting Information Fig. S8.

## Discussion

A mass continuous production strategy was introduced to realize the facile, massive production of rGO-based, 3D macrostructures. By continuously frying GO dispersion in liquid nitrogen, a large quantity of graphene oxide frozen balls (GOFB) can be synthesized. Followed by freezing drying and chemical reduction, graphene spongy balls, named GSB, can be obtained with a tunable density ranging from 1.8 mg cm^−3^ to 11 mg cm^−3^ by varying the experimental conditions. Compared with previous hydrothermal protocols^6^, this strategy has exhibited novelty and differences, which can be confirmed by several controlled experiments and further characterizations in Supporting Information (SEM and TEM images in Fig. S9, electrochemical measurements in Fig. S10, Brunauer-Emmett-Teller (BET) surface measurements and pore structure analysis in Fig. S11). In addition, a comprehensive comparison is reviewed in Table S2. Furthermore, the GSB showed good performance serving as electrodes for supercapacitor, and as adsorbents for the removal of oils and organic liquids. Satisfying performance of GSB, along with simplicity of fabrication process have demonstrated a great potential in industrial applications, such as energy storage and environment protection. Moreover, considering the feasibility of chemical modification and functionalization of GO, it is believed that this continuous, massive production strategy will have broad applications.

## Methods

### Preparation of GO dispersion

GO was synthesized from natural graphite powder using a modified Hummers method^28^. The detailed procedures could be found in ref. 6.

### Dynamic imaging of oil adsorption

Images of oil on the surface of a glass plate were taken and analyzed to characterize the dynamic process of oil adsorption. hexahydrobenzene (1.5 g) was stained with Sudan red 5B (2 mg) to facilitate the evaluation of oil adsorption.

### Electrochemical measurements

All electrochemical measurements were performed in a Solartron analytical equipment (CHI660D, Shanghai Chenhua Instruments). The GSB without any additive were pressed into film-like graphene blocks and then sandwiched between the nickel foam to form a sandwich structure as the working electrode. The loading mass of GSB was about 2 mg. The as-formed electrode was then dried at 100 °C in a vacuum oven. For three-electrode test, 6 mol L^−1^ KOH aqueous solution was used as the electrolyte. GSB, Pt wire and Ag/AgCl electrode were used as the working, counter and reference electrodes, respectively. Cyclic voltammetry and Galvanostatic charge-discharge curves were measured within the potential window range from −1.0 to 0 V. Electrochemical impedance spectroscopy was obtained in a frequency range of 0.01 Hz–10 kHz. For two-electrode symmetrical test, the working electrodes were symmetrically assembled with a non-woven fabric as separator. The specific capacitance for single electrode (*C*_*m*_, F g^−1^) was calculated from the galvanostatic charge-discharge values by using the following equation:


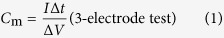



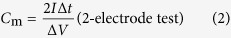


Where *I* was the current density (A g^−1^), *ΔV* refered the potential window excluding the IR drop (V) within the discharge time *Δt* (s).

The energy density (*E*, W h kg^−1^) and power density (*P*, W kg^−1^) of one electrode were calculated according to:


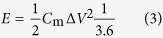



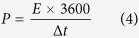


Where *C*_*p*_ represented the capacitance and *Δt* (s) was the discharge time referring to the voltage interval of the above two-symmetric-electrode test.

## Additional Information

**How to cite this article**: Wan, S. *et al.* A facile strategy for rapid preparation of graphene spongy balls. *Sci. Rep.* **6**, 32746; doi: 10.1038/srep32746 (2016).

## Supplementary Material

Supplementary Information
