# Supplementary material for: A facile strategy for rapid preparation of graphene spongy balls
Source: Sci Rep. 2016 Sep 2;6:32746. doi: 10.1038/srep32746 (PMC5009365; doi:10.1038/srep32746)
Supplement: Supplementary Information [file srep32746-s1.doc]

Supporting Information for

A facile strategy for rapid preparation of graphene spongy balls

Shu Wan, Hengchang Bi, Xiao Xie, Shi Su, Kai Du, Haiyang Jia, Tao Xu, Longbing He, Kuibo Yin and Litao Sun*

^*^slt@seu.edu.cn

**Characterization**

X-ray photoelectron spectroscopy (XPS) data were collected on an electron spectrometer (Japan, PHI 5000 VersaProbe). Scanning electron microscopy (SEM) images were recorded on an FEI QUANTA 200 (USA) instrument. Transmission electron microscopy images were recorded on an FEI TITAN 80-300 (USA) instrument. The BET surface and pore structure analysis was conducted by Micromeritics ASAP2010 (USA).


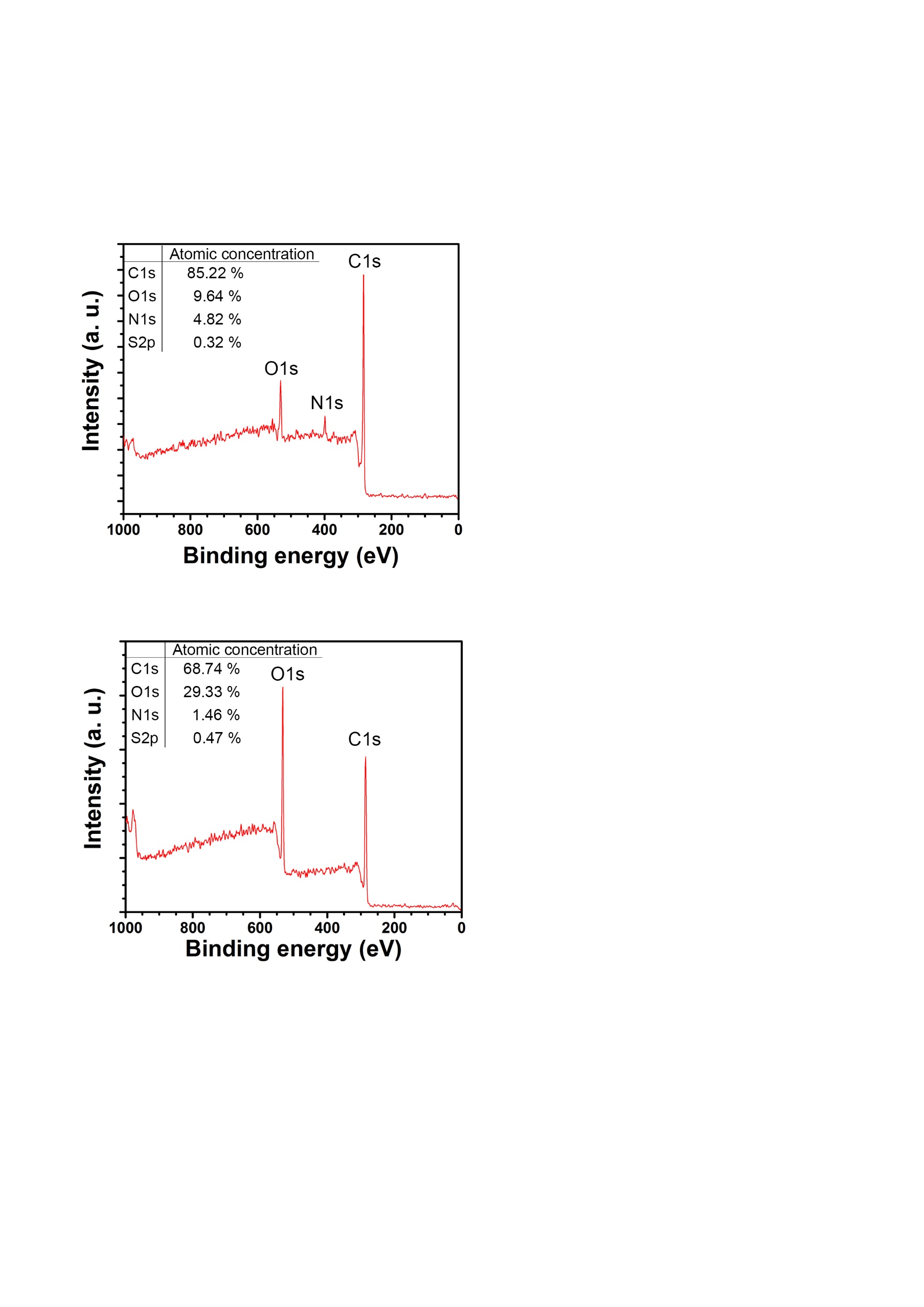


**Figure S1**. XPS spectrum for GOSB. The atomic concentration for C1s and O1s was 68.74 %, 29.33 %, respectively. (The rest: N1s 1.46 %, S2p 0.47 %).


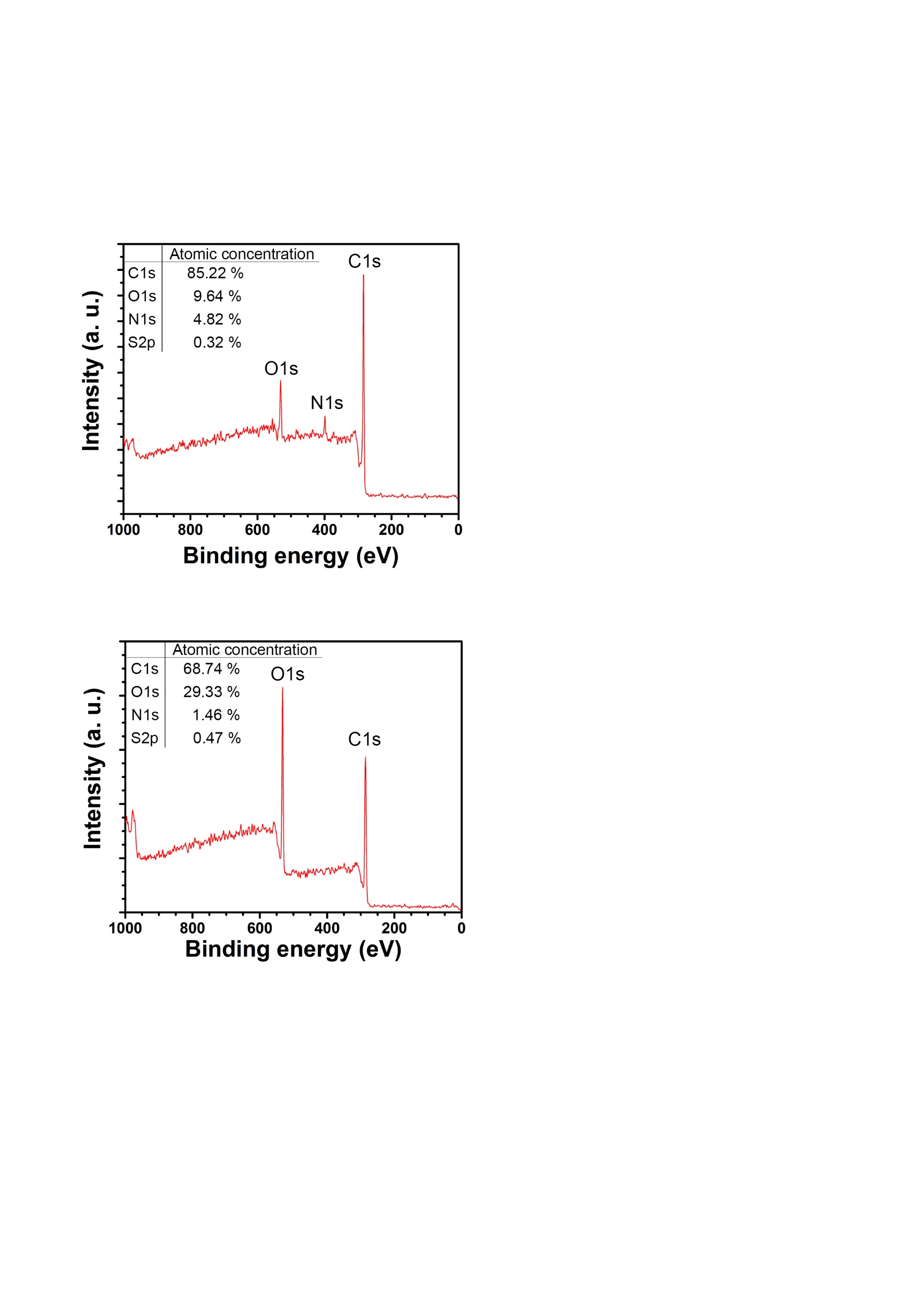


**Figure S2.** XPS spectrum for GSB. The atomic concentration for C1s and O1s was 85.22 %, 9.64 %, respectively. (The rest: N1s 4.82 %, S2p 0.32 %).


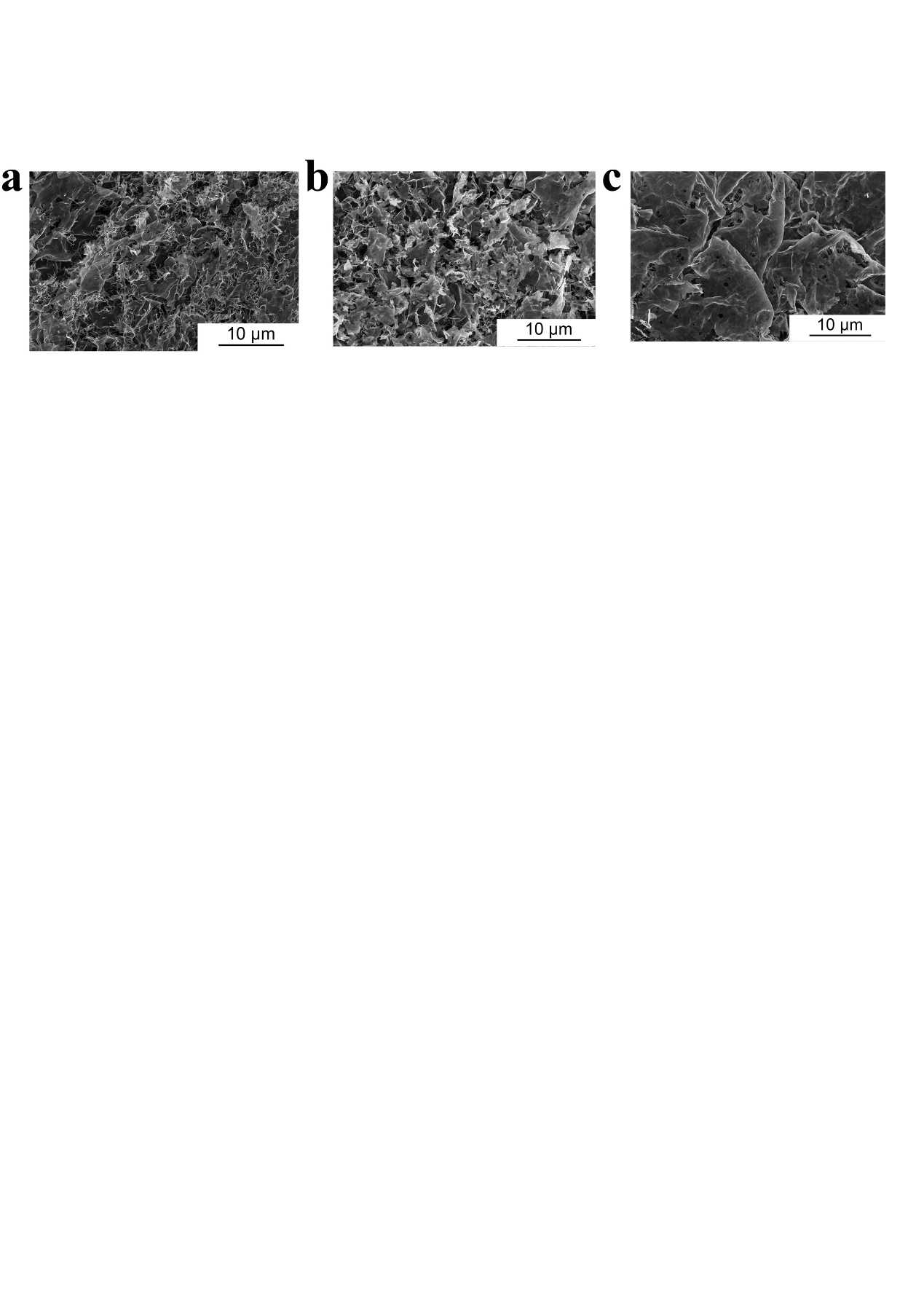


**Figure S3.** SEM images for GSB with different densities. (a)-(c) shows the surface morphology for GSB of 1.8 mg cm^-3^, 5.2 mg cm^-3^, and 10 mg cm^-3^, respectively.

Three kinds of GSB with different densities of 1.8, 5.2, and 10 mg cm^-3^ were used to study the mechanical properties of GSB. Namely, three samples were denoted as GSB-1, GSB-2, and GSB-3, respectively. The experiment setup was schematically demonstrated in Figure S4a. A mechanized step motor z-axis stage (Mark-10, ESM 301, 0.01 mm resolution) along with a force gauge (Mark-10 M5, 1 g resolution) were adopted to supply the press load to the samples, and a laptop (Dell XPS 13) were connected to the force gauge by a cable to collect data. The relationship between the strain and the applied force was plotted in Figure S4b. All the three curves showed similar slope value before the applied force reached 0.15 N. It seemed that elastic deformation mainly took place in this regime. When applied force over 0.15 N, plastic deformation happened. In this regime, GSB-3 exhibited the highest slope value among three samples also the slope value had the positive relationship with sample density. Eventually, all three samples were squashed and the highest force each sample received were 0.282, 0.362, and 0.812 N, respectively. It was obvious that sample with higher density exhibiting better mechanical performance.


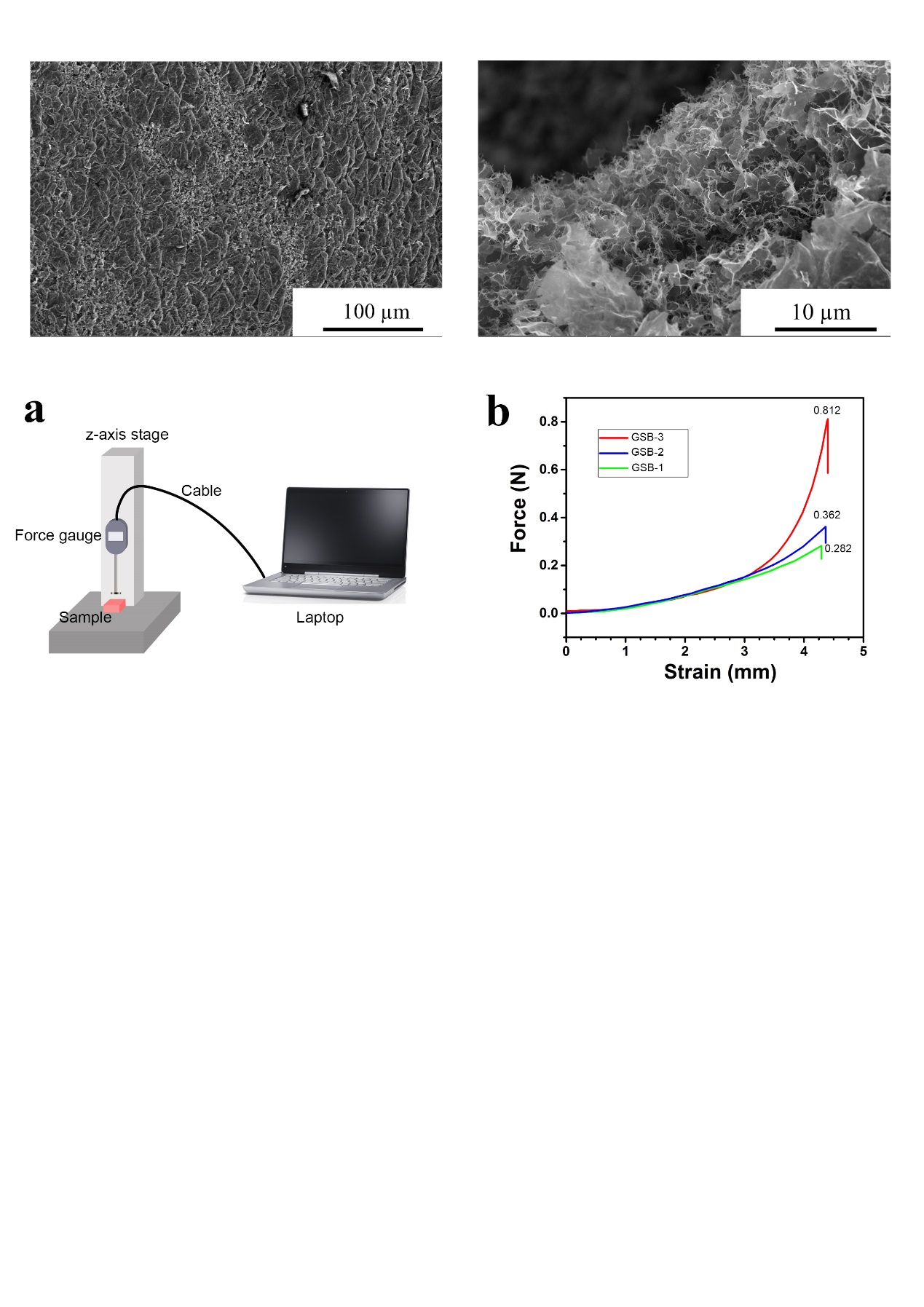


**Figure S4.** (a) The schematic illustration of experiment setup. (b) The mechanical properties of GSB with different densities.


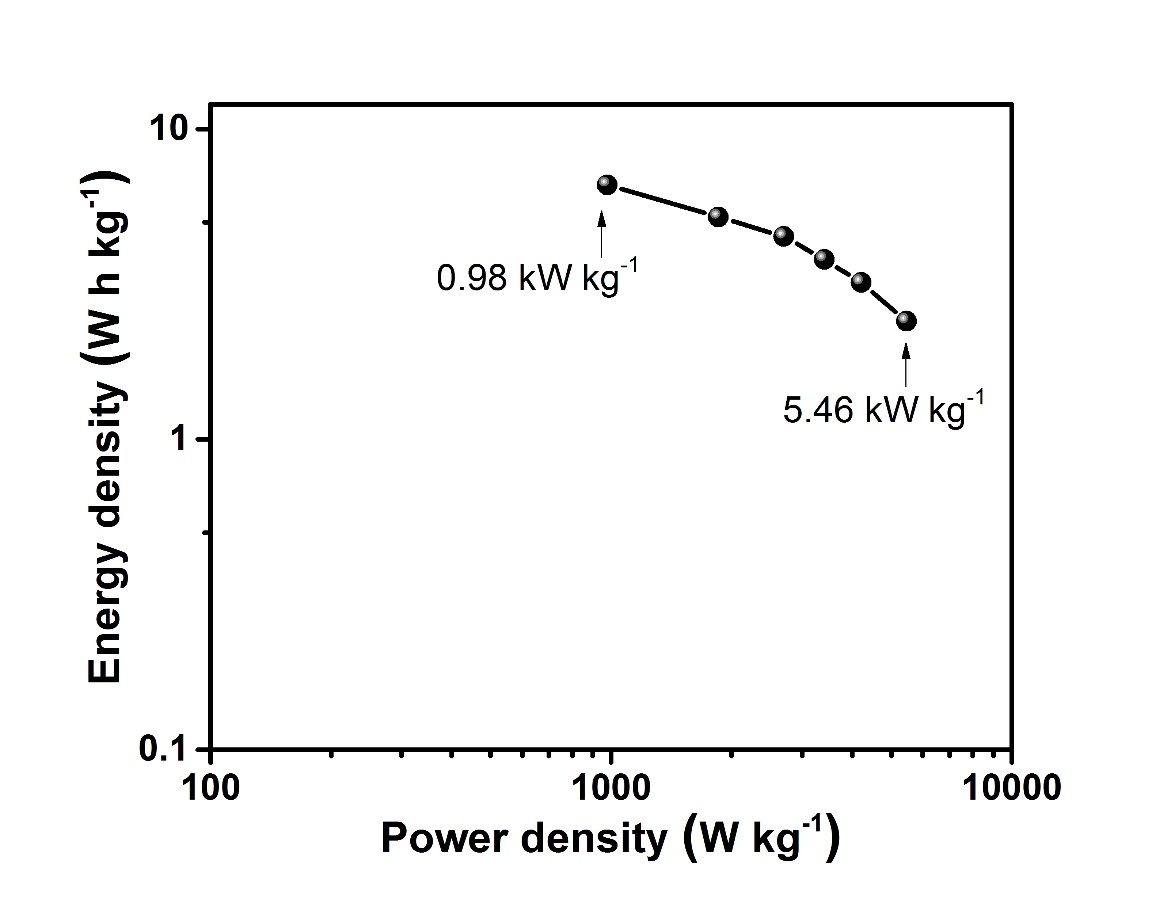


**Figure S5.** Ragone plot (Energy density and power density) of GSB.


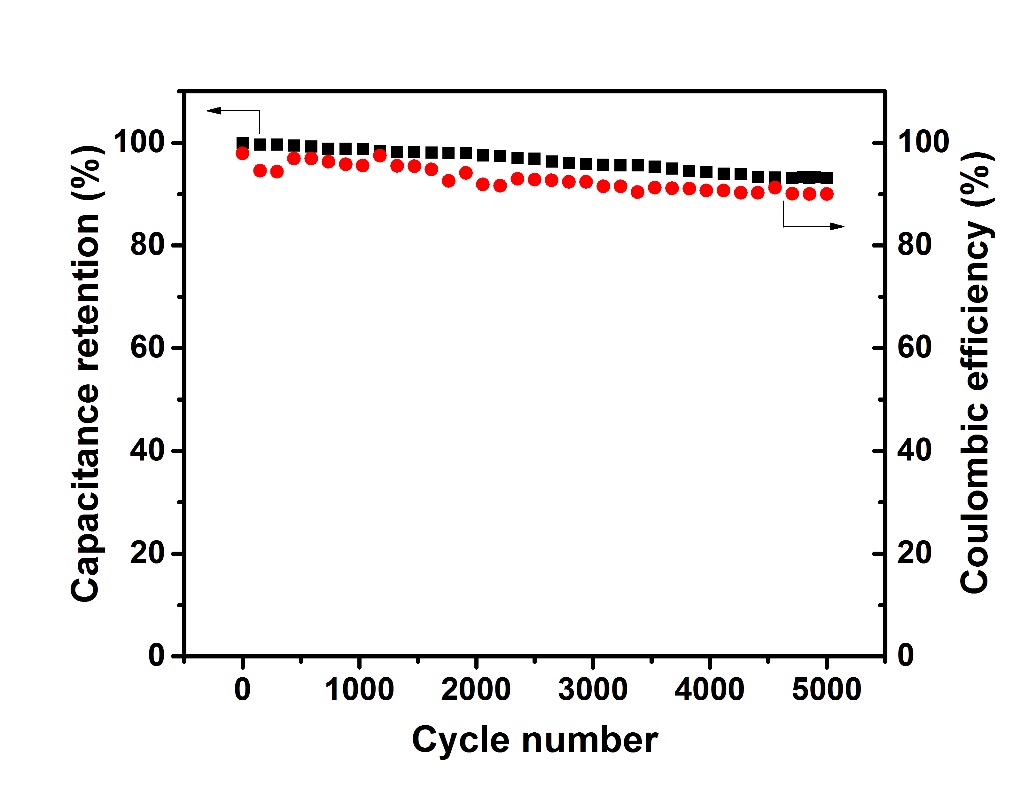


**Figure S6.** The recycle ability and coulombic efficiency of GSB.

A comparison table of 3D graphene based supercapacitor with the state of art in recent years is presented in Table S1.

**Table S1.** Comparison of 3D graphene-based supercapacitors in recent years

| Electrode | Specific capacitance (F g^-1^) | | Electrolyte | References |
| --- | --- | --- | --- | --- |
| Holey graphene nanosheets | 197 (1 A g^-1^, 3E) | 6 M KOH | | 2 |
| Reduced graphite oxide with high density | 182 ( 1 Ag^-1^, 2E) | 6 M KOH | | 3 |
| Reduced graphene oxide templated by CaCO_3_ spheres | 201 (0.1 A g^-1^, 2E) | 1 M H_2_SO_4_ | | 4 |
| Hollow graphene spheres | 193 (1 A g^-1^, 2E) | 6 M KOH | | 5 |
| rGO foam | 110 (0.5 A g^-1^, 2E) | 1 M H_2_SO_4_ | | 6 |
| Graphene on nickel foams  Nitrogen-rich graphene nanosheets | 164 (10 mV s^-1^, 3E)  201 (0.05 A g^-1^, 3E) | 6 M KOH  1 M H_2_SO_4_ | | 7  8 |
| Porous graphene macroform | 238 (0.1 A g^-1^, 2E) | 6 M KOH | | 9 |
| Nanopores three-dimensional graphene | 200 (1 mV s^-1^, 3E) | 1 M NaCl | | 10 |
| GSB-based electrode | 124 (1 A g^-1^, 3E) | 6 M KOH | | this work |

3E: three-electrode test; 2E: two-electrode test.


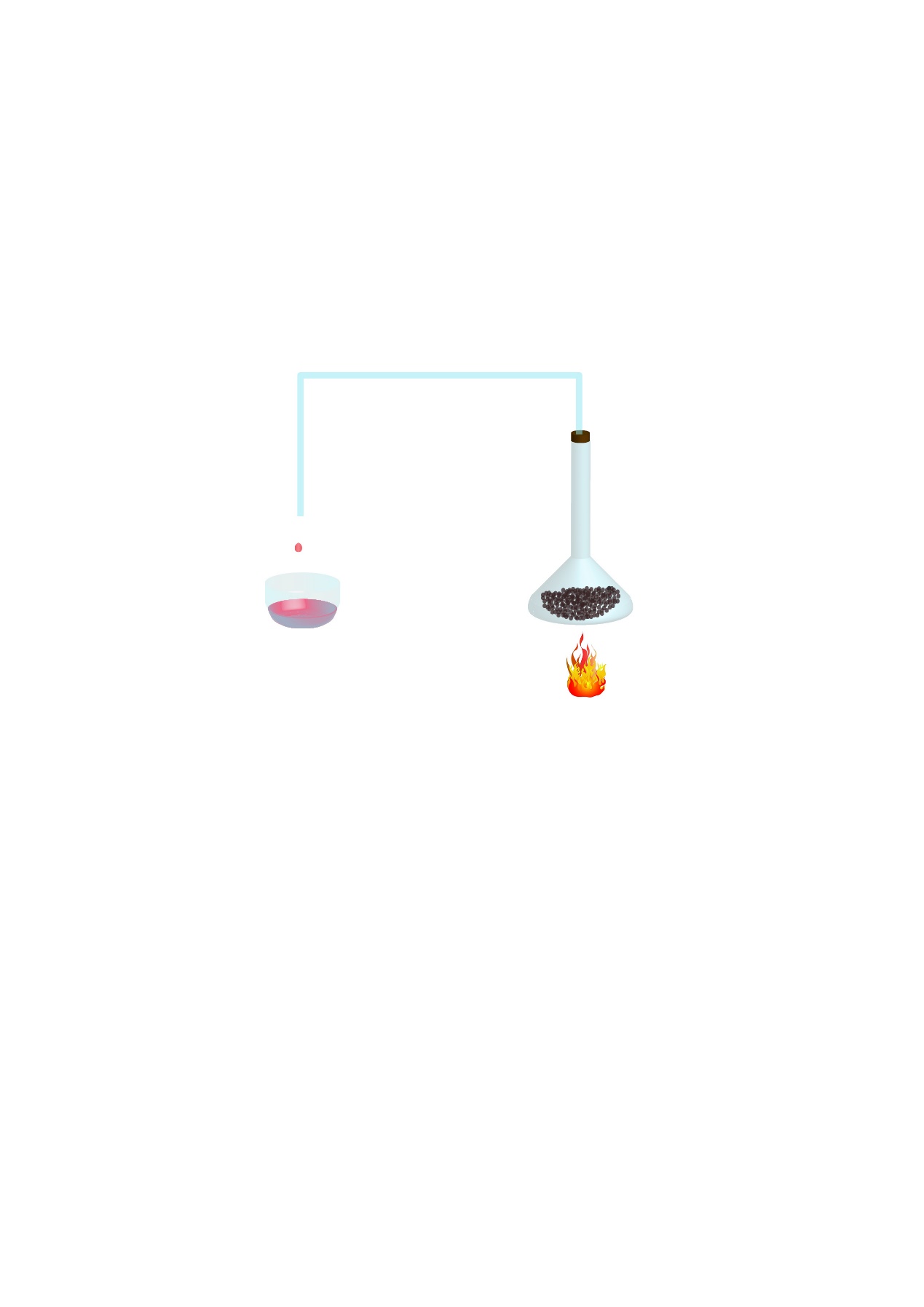


**Figure S7.** Typical experiment setup for distillation.


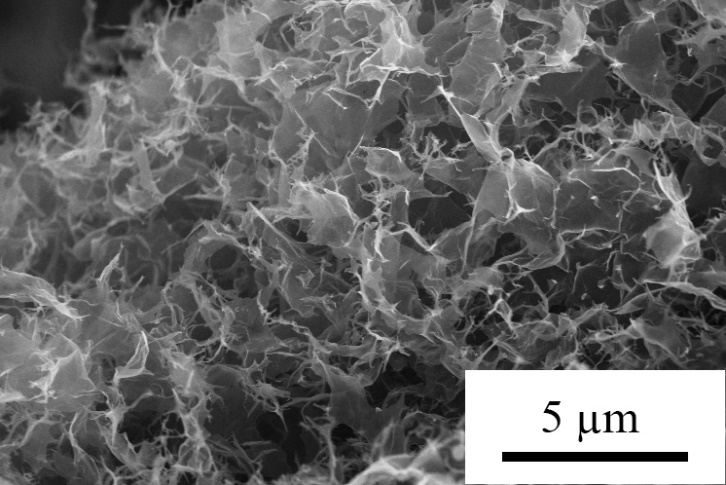


**Figure S8.** SEM image of GSB after distillation.

The detail microstructure information of graphene sponge (GS) was described in Figure S9 through SEM and TEM. From the SEM images (Figures S9a and S9b), it could be found that the sponge was made of graphene platelets, which was less than 10 layers proven by TEM images (Figures S9c and S9d). The structure morphology was quite different from that of GSB. Compared with GSB, the average pore size was much bigger and the wall of each pore was thicker and denser. It seemed that the aggregation in hydrothermal process ascribed to the structure difference. In a typical hydrothermal process, dispersed graphene oxide sheets would be reduced to graphene sheets due to the high temperature and high pressure, resulting the self-assembly process therefore the aggregation of graphene sheets.^1^ The aggregation would lead to the contraction of prepared sponge monolith (up to 90% reduction by volume of the former GO dispersion).

On the contrary, the shaping process of GSB was not caused by the chemical reaction. It was the physical squeezing during ice nuclei growth that formed the microstructure and the spongy ball-like shape further. The ‘frying’ process did not change the volume of the sponge compared to the GO dispersion. Therefore the density of the monolith was similar to the concentration of GO dispersion. Based on this consideration, liquid nitrogen frying enabled a lighter sponge than hydrothermal reaction process.


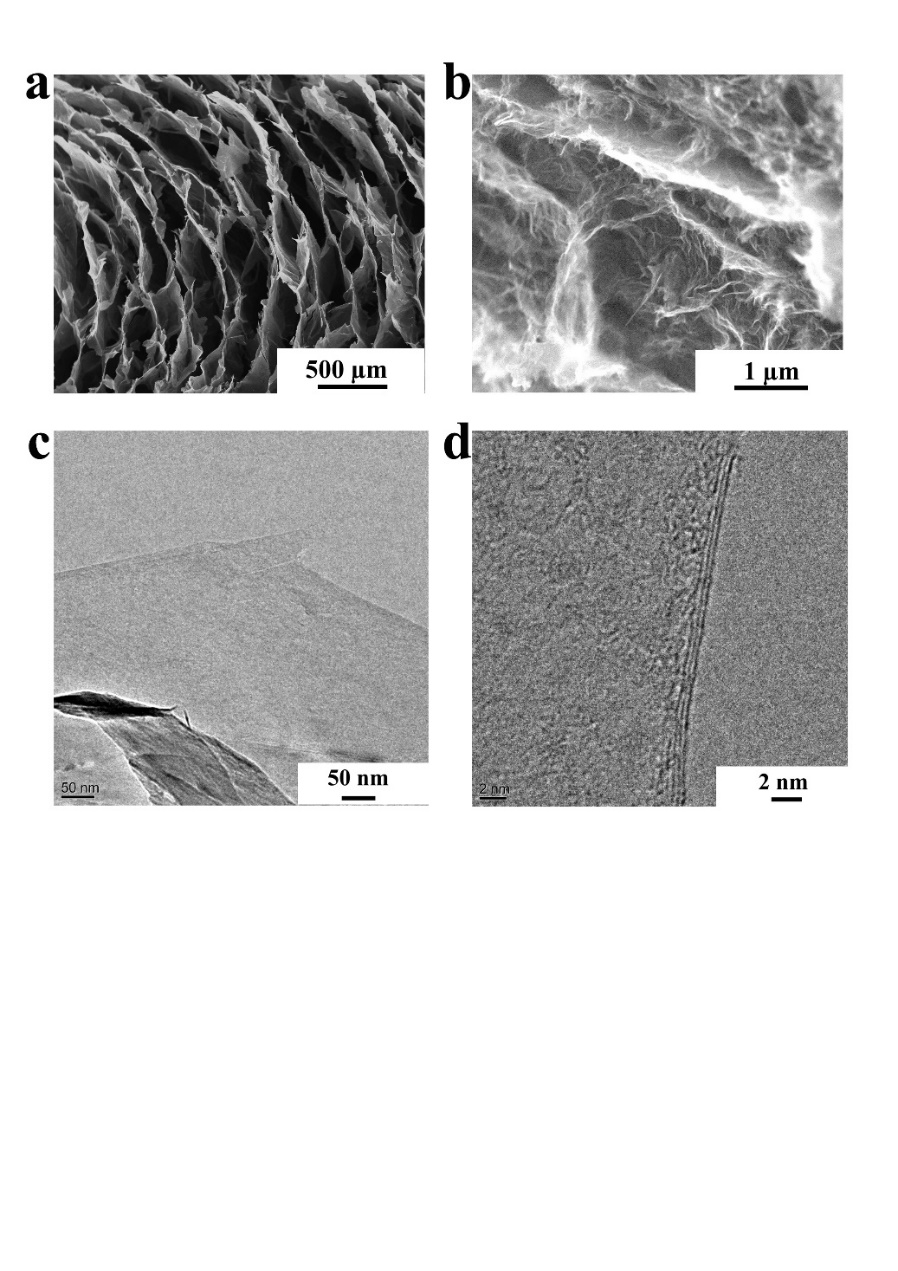


**Figure S9.** Microstructure of graphene sponge produced by hydrothermal method. (a) (b) SEM images of graphene sponge. (a): Low magnification image. (b): High magnification image. (c) (d) TEM images of graphene sponge. (c): Low magnification image. (d): High magnification image.

The electrochemical performance of GS electrodes was analyzed by cyclic voltammetry (CV) and galvanostatic charge/discharge measurements under the same equipment and the results were shown in Figure S10. The overall performance was better than that of GSB. The specific capacitance was 235 F g^-1^, which was higher than that of GSB. This result is in accordance with the Brunauer-Emmett-Teller (BET) surface and pore structure analysis, which was shown in Figure S11. In Figure S11a, GS exhibited a Type I isotherm together with some characteristics for Type IV. In contrast, GSB did not show a similar isotherm and pore size distribution (PSD) curves (Fig. S11b) exhibited that GSB had less mesopores than GS. In addition, compared with surface area of graphene sponge (387.6 m^2^ g^-1^), the value of GSB (83.2 m^2^ g^-1^) was much smaller. The differences in BET surface and electrochemical performance were attributed to different structure produced by shaping strategies. Despite of the aggregation of graphene sheets, it seemed that the self-assembly during hydrothermal reaction still remained plenty of mesopores.


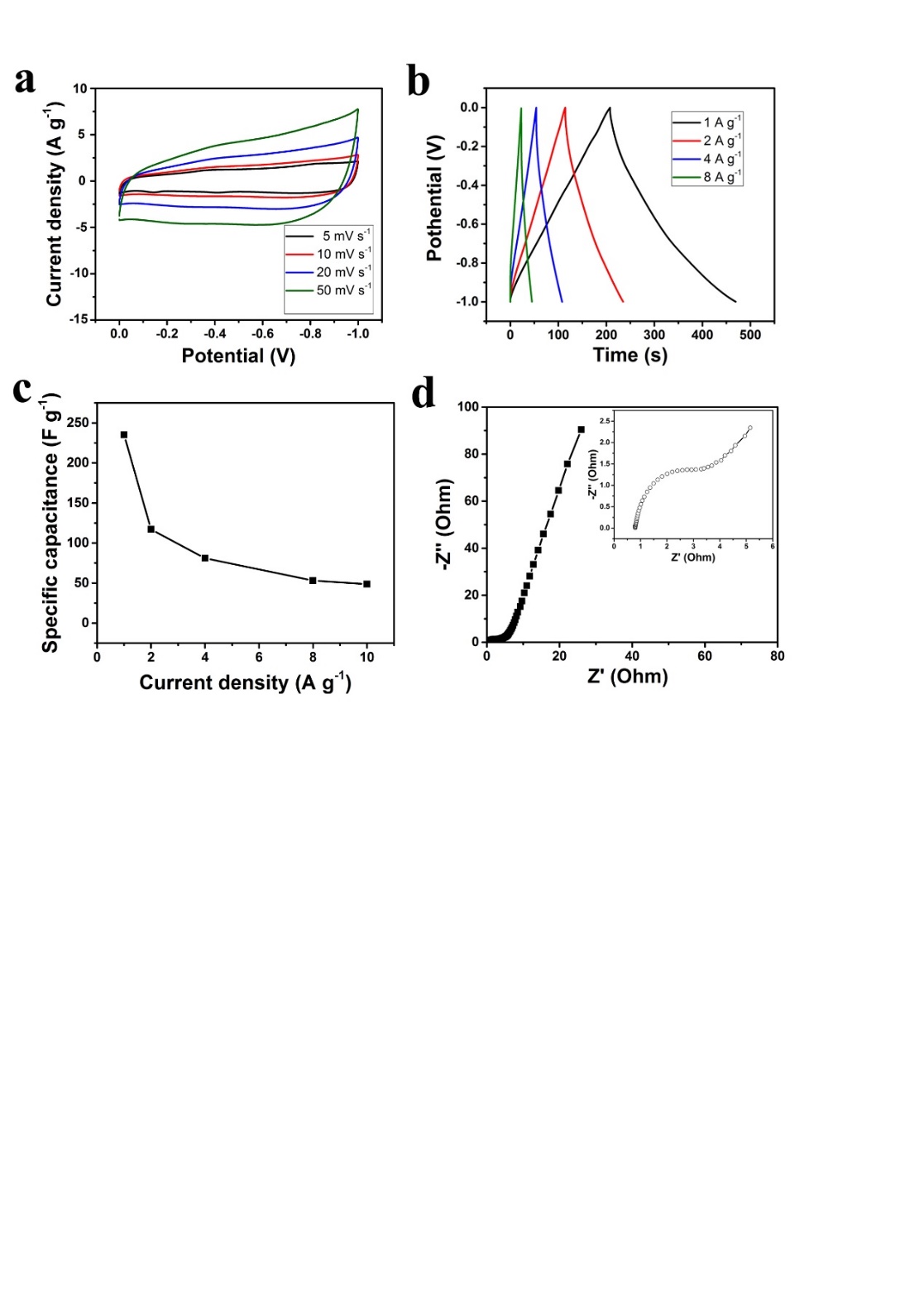


**Figure S10.** Performance of graphene sponge-based supercapacitors. (a) The CV curves at different scan rates from 5 to 50 mV s^-1^. (b) Galvanostatic charge/discharge measurements at different current densities. (c) Specific capacitances at different current densities. (d) Electrochemical impedance spectroscopy at different frequencies, ranging from 0.01 Hz to 10 kHz.


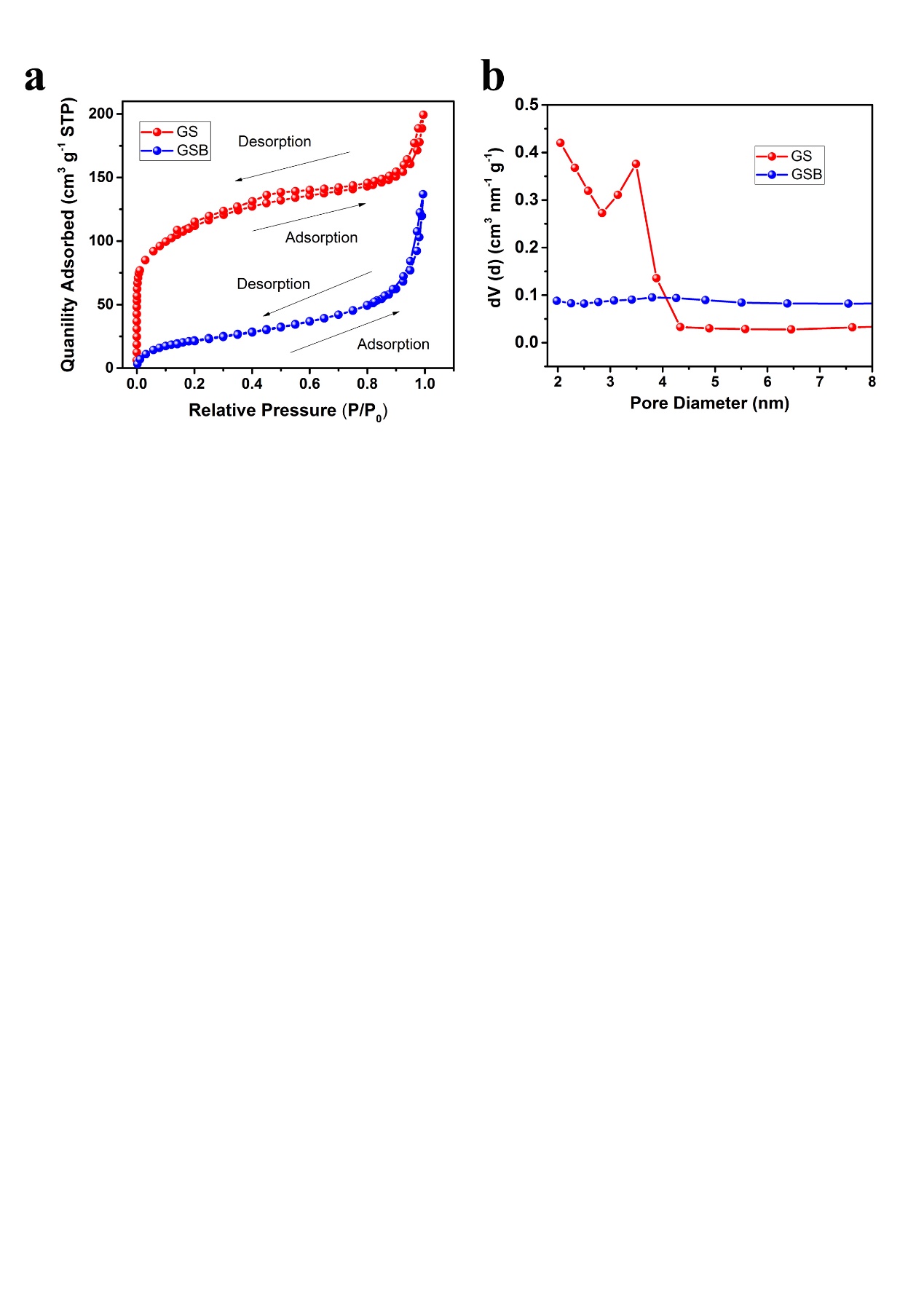


**Figure S11.** The BET surface and pore structure analysis for graphene sponge (GS) graphene sponge ball (GSB). (a) Nitrogen adsorption isotherms and (b) Pore size distributions (PSD) of GS and GSB.

A comprehensive comparison between graphene sponge (GS) and graphene sponge ball (GSB) was exhibited in Table S2. Actually, the GSB did not show better performance in energy storage as porous electrodes for supercapacitors and higher BET value. However, GSB had advantage when used as adsorbents for oils and organic solvents. Moreover, the synthesis process of GSB was much more facile than that of GS. Only 30 seconds were needed to shaping GSB in the present work and there was no specialized requirements on the container in synthesis process. Therefore, the ‘frying’ strategy seemed suit for the massive, continuous production in industrial production better than the hydrothermal protocol.

**Table S2.** Comparison between GSB and graphene sponge

| Synthesis strategy | Liquid N_2_ fired method (GSB) | Hydrothermal reaction (GS) |
| --- | --- | --- |
| Assembly time | <30 s | ~10 h |
| Special equipment | None | Autoclave |
| Production mode | Continuous | Batch limited |
| Adsorption of graphene spongy (of its own weight) | 40~105.4 | 22~86^11^ |
| Surface area (BET) of graphene spongy (m^2^ g^-1^) | 83.2 | 387.6 |
| Specific capacitance of graphene spongy (F g^-1^) | 124 | 235 |

**References**

1. Bi, H. *et al.* Low Temperature casting of graphene with high compressive strength. *Adv. Mater*. **24**, 5124-5129 (2012).
2. Peng, Y. Y. *et al*. A facile approach to produce holey graphene and its application in supercapacitors. *Carbon* **81**, 347 -356 (2015).
3. Li, Y. & Zhao, D. Preparation of reduced graphite oxide with high volumetric capacitance in supercapacitor. *Chem. Commum*. **51**, 5598-5601 (2015).
4. Gu, Y., Wu, H., Xiong, Z., Abdulla, W. A. & Zhao, X. S. The electrocapacitive properties of hierarchical porous reduced graphene oxide templated by hydrophobic CaCO_3_ spheres. *J. Mater. Chem. A* **2**, 451- 459 (2014).
5. Cao, J. *et al*. Hollow graphene spheres self-assembled from graphene oxide sheets by a one-step hydrothermal process. *Carbon* **56**, 383-391(2013).
6. Niu, Z., Chen, J., Hng, H. H., Ma, J. & Chen, X. A leavening strategy to prepare reduced graphene oxide foams. *Adv. Mater.* **24**, 4144-4150 (2012).
7. Chen, Y., Zhang, X., Yu, P. & Ma, Y. Electrophoretic deposition of graphene nanosheets on nickel foams for electrochemical capacitors. *J Power Sources* **195**, 3031-3035 (2010).
8. Wen, Y., Rufford, T. E., Hulicova-Jurcakova, D., Zhu, X. & Wang, L. Structure control of nitrogen-rich graphene nanosheets using hydrothermal treatment and formaldehyde polymerization for supercapacitors. *ACS Appl. Mater. Interfaces* **8**, 18051-18059 (2016).
9. Tao, Y. *et al*. Towards ultrahigh volumetric capacitance: graphene derived highly dense but porous carbons for supercapacitors. *Sci. Rep*. **3**. 2975-2982 (2013).
10. Shi, W. *et al*. Ultrahigh performance of novel capacitive deionization electrodes based on a three-dimensional graphene architecture with nanopores. *Sci. Rep.* **6**. 18966-18974 (2016).
11. Bi, H. *et al.* Spongy graphene as a highly efficient and recyclable sorbent for oils and organic solvents. *Adv. Func. Mater.* **22**, 4421-4425 (2012)
